# Reproducibility of APT-weighted CEST-MRI at 3T in healthy brain and tumor across sessions and scanners

**DOI:** 10.1038/s41598-023-44891-0

**Published:** 2023-10-23

**Authors:** Yulun Wu, Tobias C. Wood, Sophie H. A. E. Derks, Ilanah J. Pruis, Sebastian van der Voort, Sophie E. M. Veldhuijzen van Zanten, Marion Smits, Esther A. H. Warnert

**Affiliations:** 1 Department of Radiology & Nuclear Medicine, Erasmus MC, Rotterdam, The Netherlands; 2 Brain Tumour Centre, Erasmus MC Cancer Institute, Rotterdam, The Netherlands; 3 Department of Neuroimaging, Institute of Psychiatry, Psychology & Neuroscience, King’s College London, London, UK; 4 Department of Medical Oncology, Erasmus MC-University Medical Centre Rotterdam, Rotterdam, The Netherlands; 5 Medical Delta, Delft, The Netherlands

**Keywords:** Biomarkers, Biomedical engineering

## Abstract

Amide proton transfer (APT)-weighted chemical exchange saturation transfer (CEST) imaging is a recent MRI technique making its way into clinical application. In this work, we investigated whether APT-weighted CEST imaging can provide reproducible measurements across scan sessions and scanners. Within-session, between-session and between scanner reproducibility was calculated for 19 healthy volunteers and 7 patients with a brain tumor on two 3T MRI scanners. The APT-weighted CEST effect was evaluated by calculating the Lorentzian Difference (LD), magnetization transfer ratio asymmetry (MTR_asym_), and relaxation-compensated inverse magnetization transfer ratio (MTR_REX_) averaged in whole brain white matter (WM), enhancing tumor and necrosis. Within subject coefficient of variation (COV) calculations, Bland–Altman plots and mixed effect modeling were performed to assess the repeatability and reproducibility of averaged values. The group median COVs of LD APT were 0.56% (N = 19), 0.84% (N = 6), 0.80% (N = 9) in WM within-session, between-session and between-scanner respectively. The between-session COV of LD APT in enhancing tumor (N = 6) and necrotic core (N = 3) were 4.57% and 5.67%, respectively. There were no significant differences in within session, between session and between scanner comparisons of the APT effect. The COVs of LD and MTR_REX_ were consistently lower than MTR_asym_ in all experiments, both in healthy tissues and tumor. The repeatability and reproducibility of APT-weighted CEST was clinically acceptable across scan sessions and scanners. Although MTR_asym_ is simple to acquire and compute and sufficient to provide robust measurement, it is beneficial to include LD and MTR_REX_ to obtain higher reproducibility for detecting minor signal difference in different tissue types.

## Introduction

Amide proton transfer (APT)-weighted chemical exchange saturation transfer (CEST) imaging is a recent MRI technique making its way into clinical application. The APT CEST signal is sensitive to amide protons that resonate at 3.5 ppm^1^. After an off-resonance saturation pulse is given at 3.5 ppm, a saturation transfer from exchangeable amide protons of endogenous mobile proteins and peptides to surrounding water cause a reduction of the bulk water signal, which is called the CEST effect^2^. APT-weighted CEST has shown great potential for clinical glioma imaging, including predicting IDH mutation status for diagnosis^3,4^, response assessment to treatment^5–7^, and predicting overall and progression-free survival^8^.

Clinical translation of this technique is coming closer with the recently published consensus parameters for brain tumor imaging^9^. Hence reproducibility of the technique is important, for separating healthy and diseased tissue or for follow-up of tumors over time. To evaluate APT-weighted CEST in glioma diagnostics, studies proposed to apply magnetization transfer ratio asymmetry (MTR_asym_) to minimize the influence of broad magnetisation transfer (MT) effects^9–13^. MTR_asym_ is easy to compute and requires sampling of only a few frequency offsets during imaging acquisition. However, saturation pools which influence the spectrum on opposite sides of the main resonance frequency cannot be independently evaluated by MTR_asym_; for example the effect of APT (3.5 ppm) and nuclear Overhauser enhancement (NOE, at − 3.5 ppm) are both reflected in MTR_asym_ at 3.5 ppm. Additionally, the NOE effect can give a different contrast in tumor imaging than the APT effect^14,15^. Thus it is beneficial to separate the APT from the NOE signal by advanced metrics, namely Lorentzian difference (LD) via multi-pool Lorentzian fitting^16–22^ and relaxation-compensated inverse magnetization transfer ratio (MTR_Rex_^23,24^) to account for spillover effects that cannot be compensated by LD analysis^17,18,25–28^.

Previous research shows high reproducibility of APT-weighted imaging. However this work focused on APT evaluated only by MTR_asym_^29–31^, or evaluated by advanced metrics but with a very limited sample size (N = 3)^14^, or at ultra-high field strength^32^. To date, few studies have evaluated APT-weighted imaging by LD or MTR_REX_ metrics at 3 Tesla. In addition, for measurement of patient in different hospitals, measurement of reproducibility between different scanners is also an important aspect for clinical translation of APT-weighted imaging.

In this work, we investigated the repeatability and reproducibility of APT-weighted CEST imaging in healthy volunteers and patients with a brain tumor by evaluating LD, MTR_asym_ and MTR_REX_. To give a comprehensive overview of reproducibility of APT-weighted CEST MRI, we included measurements of within session (repeatability) and between sessions (reproducibility), as well as between two different scanners (reproducibility).

## Methods

### Participants

This study was approved by the medical ethics committee of the Erasmus MC, Rotterdam, the Netherlands, and performed in accordance with the declaration of Helsinki. Nineteen healthy volunteers were included (age range = 19–62 years, median age = 25 years, gender = 7 males/12 females) who provided written informed consent to have their imaging data used for the study. In addition, seven patients with primary or recurrent malignant brain tumors (high-grade glioma, N = 5; brain metastasis, N = 2) were included. These patients underwent repeated PET-MRI as part of the Passage Study at Erasmus MC (NL73457.078.20) and provided written informed consent to have their imaging data used for research purposes. Patient characteristics are shown in Table 1.

**Table 1:** Patient characteristics.

Patient	Age	M/F	Tumor type
1	70	M	(residual) Brain metastasis from lung adenocarcinoma
2	73	M	(residual) Glioblastoma WHO grade 4, IDH wildtype
3	61	M	glioblastoma WHO grade 4, IDH wildtype
4	69	M	(residual) Glioblastoma WHO grade 4, IDH wildtype
5	57	M	(residual) Brain metastasis from lung adenocarcinoma
6	62	F	(residual) Glioblastoma WHO grade 4, IDH wild type
7	72	M	(residual) Oligodendroglioma WHO grade 4, IDH mutated 1p/19q co-deleted

### MRI experiment

A 3 Tesla MRI scanner equipped with a 32-channel head coil (MR750, General Electric, Chicago, USA) was used for the within/between session comparisons in healthy volunteers. A 3 Tesla SIGNA PET-MRI scanner with a 24-channel head coil (General Electric, Chicago, USA) was used to assess between-scanner reproducibility in healthy volunteers and between session reproducibility in patients. One scan session contained at minimum a T_1_-weighted structural scan and 2 identical CEST scans. The total scan duration of one session was approximately 15 min.

**Figure 1:**
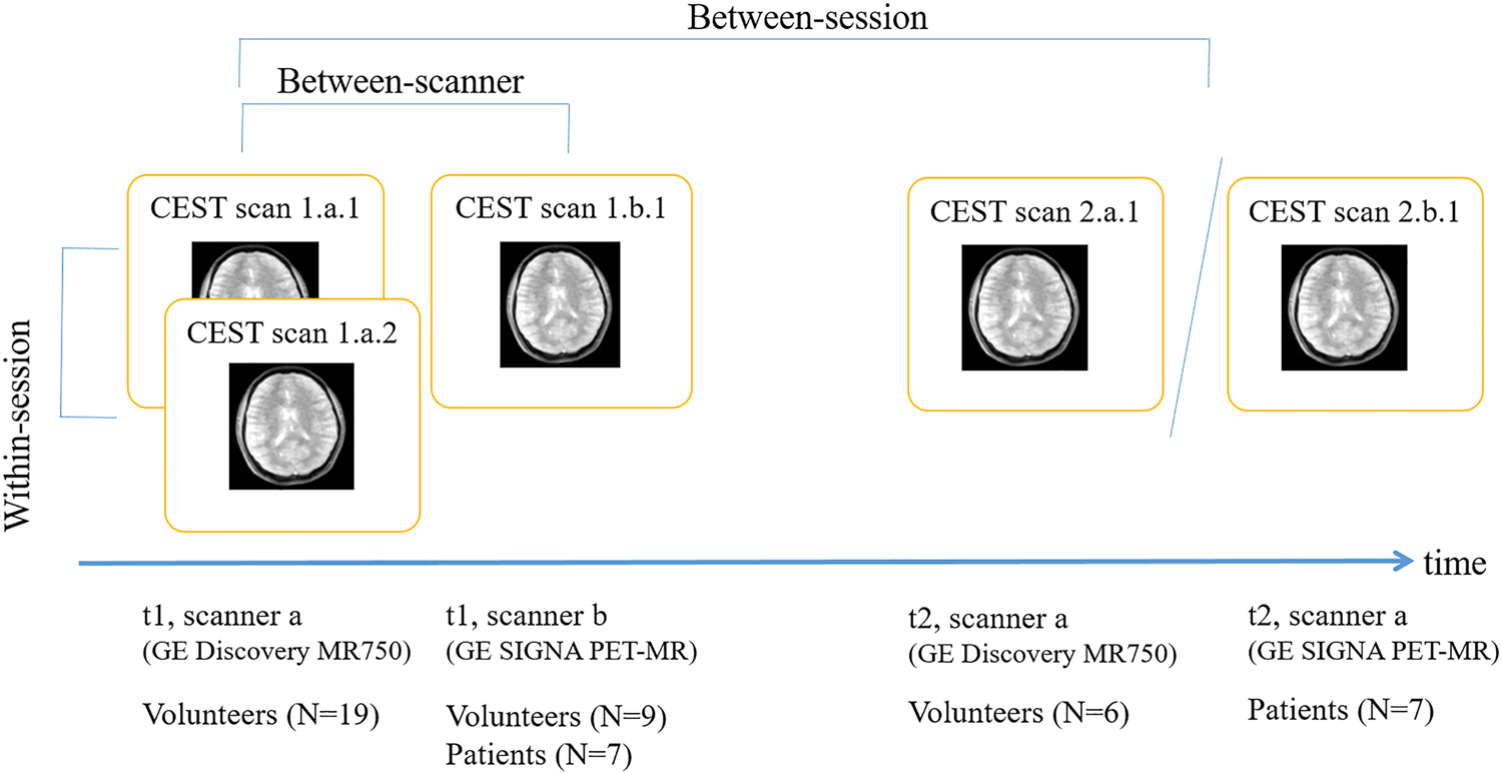
Description of reproducibility experiments, including within-session, between-session and between-scanner reproducibility. For healthy volunteers, time between t = 1 and t = 2 was 7 days. For patients, the median time interval was 4 days.

The design of this study is presented in Fig. 1. To assess within-session repeatability, each volunteer (N = 19) underwent one scan session including two identical CEST scans. To assess between-session reproducibility, six volunteers underwent the same session one week after the first session was acquired. To assess between scanner reproducibility, we applied one scan session per scanner for each nine volunteer on the same day. For the patients (N = 7), only between session reproducibility (on the same scanner) was assessed. For patients, the median time interval between 1.b.1 and 2.b.1 was 4 days.

### Image acquisition

The pulse sequences used for the following imaging acquisition were identical for both systems included. A 3D snapshot CEST image acquisition^14^ was used with the following parameters: slice thickness = 3 mm, 14 slices, in-plane resolution 1.7 $\times$ 1.7 mm^2^, matrix size = 128 $\times$ 104, read out flip angle 6°, ASSET acceleration factor of 3. The field of view was manually placed with the top slices 20 mm above the corpus callosum for suitable tissue separation of white and grey matter (WM respectively GM). Saturation was performed with B_1,RMS_ = 1.5 µT and with 80 Gaussian shaped pulses of 20 ms with 50% duty cycle. Z-spectra were obtained for 43 frequency offsets distributed between − 100 and 100 ppm, relative to the water resonance set to 0 ppm (at ± 100 ppm, ± 50 to ± 10 ppm in steps of 10 ppm, ± 9 to ± 5 ppm in steps of 1 ppm, ± 4.5 to ± 1 in steps of 0.5 ppm, and − 0.5 to 0.5 ppm in steps of 0.25 ppm). Four images were obtained with saturation pulses at − 300 ppm, and the last of four images were selected as the reference images, yielding a total time of 4:30 min for each CEST scan.

A high resolution T_1_-weighted structural image was acquired for anatomical reference. In healthy volunteers, a 3D FSPGR sequence was acquired, TE = 2.1 ms, TR = 6.1 ms, voxel size = 1.0 × 1.0 × 0.5 mm^3^, field of view 256 mm, 352 slices. In patients, a 3D FSPGR sequence was acquired (TE = 3.1 ms, TR = 7.7, voxel size = 0.9 × 0.9 × 1.6 mm^3^, field of view 256 mm, 228 slices) both before and after injection of a total of 15 ml gadolinium-based contrast agent (GBCA; Gadovist®, gadobutrol 1 mmol/ml, Bayer AG, Berlin, Germany). The CEST scans were acquired prior to injection of GBCA.

### Data analysis

Image analysis was done with in-house written Matlab scripts (R2021a, The MathWorks, Natick, USA)^33^ and the freely available FMRIB Software Library (FSL 5.0, Oxford, UK)^34,35^. The CEST contrast maps of the brain were generated based on routines described in Wu et al.^15^. Z-spectra were calculated from saturated CEST images normalized by the reference image. In each voxel, two-pool Lorentzian fitting was performed to fit the direct water saturation (DS) and MT effect to the Z-spectra. LD was computed to evaluate the CEST effect by subtracting the Z-spectra from the fitted Lorentzian function and LD at 3.5 ppm was used for APT-weighted imaging. After that, the shift between the minimum value of Lorentzian fitting and 0 ppm was recorded to create the B0 inhomogeneity map. This map was applied on Z-spectra and LD for voxel-wise B_0_ correction to compensate for local field inhomogeneity. Subsequently, MTR_asym_ and MTR_REX_^23,24^ were computed for APT at + 3.5 ppm.

In the healthy volunteers, WM, GM and the cerebrospinal fluid in the lateral ventricles (CSF) were selected as regions of interest (ROI). These ROIs were segmented on the high resolution T_1_-weighted structural scans by 'FAST', available within the free online software FMRIB Software Library (FSL) v6.0^34,35^. In the patients, the contrast enhancing area of tumor (CE), the area(s) encompassed by the enhancement (necrotic core), and contralateral healthy WM were selected as ROIs. Tumors were segmented using an in-house segmentation pipeline. First, pre-, and post-contrast T_1_-weighted, T2-weighted and FLAIR scans were rigidly groupwise registered to the post-contrast T1-weighted space. Rigid registration was followed by an affine registration, to the ICBM 152 2009a nonlinear symmetric atlas, using Elastix (version 5.0.1)^36–38^. Automatic segmentation was then performed using HD-GLIO^39,40^, nnUNet task 1 and 82, and an extended version of nnUNet^40,41^. HD-GLIO is a segmentation algorithm specifically designed for enhancing glioma and is available at https://github.com/NeuroAI-HD/HD-GLIO. Segmentation predictions were combined using the multi-label STAPLE algorithm^42^. The segmentations of the tumor contrast enhancing (CE) area and necrotic core were visually inspected and manually corrected if needed, using ITK-SNAP version 3.6.0 (University of Pennsylvania and Utah, USA)^43^. To register ROIs generated in the T_1_-weighted-space of a participant to the CEST space, linear transformations were performed by registering the CEST image acquired at 6 ppm into T1-weighted space with ‘FLIRT’, available within FSL. The inverse of this transformation was used to register the ROIs from the T_1_-weighed-space to the CEST space.

### Statistical analysis

Per participant and per ROI, the average APT-weighted LD, MTR_asym_ and MTR_REX_ were calculated. To assess within-session repeatability and between-session and between scanner reproducibility, the coefficient of variation (COV) and Bland–Altman plots were generated.

The calculation of COV was based on previous methods^44^. In each participant, the COV was calculated by dividing the standard deviation by the absolute mean of each APT-weighted metric per ROI across the different measurements: within session, between sessions, between scanners, and all sessions. The equations used for these calculations are given in the Appendix. Unless otherwise stated, the group median and interquartile ranges [Q1–Q3] for the COV are reported.

Bland–Altman plots were created by plotting the ROI averages against the differences between the two measurements per participant used to assess within-session repeatability and between- session and between-scanner reproducibility for each CEST metric.

To test whether there were any significant differences between APT-weighted CEST measurements at different moments/scanners, linear mixed effect models were applied to investigate the effects of within session, between sessions and between scanners variation on the CEST measurements.

Statistical analysis was performed with R studio v2022.2.1.461^45^ and Microsoft Excel 2016. The level of statistical significance was set at p < 0.05.

### Ethical approval

All procedures performed in studies involving human participants were approved by the medical ethics committee of the Erasmus MC and in accordance with the 1964 Helsinki declaration and its later amendments or comparable ethical standards.

### Informed consent

Written informed consent was obtained from all subjects in this study.

## Results

### Healthy volunteers

The COVs are shown in Table 2. The within-session, group median COVs of LD and MTR_REX_ APT in WM (N = 19) were 0.56 [0.20–1.01]% and 0.84 [0.38–1.27]% respectively. The within-session, group median COV was larger and had larger interquartile range for APT evaluated by MTR_asym_ (2.62 [0.94–7.08] % in WM). Across scan sessions, LD APT and MTR_REX_ APT showed consistently low COVs within session, between session and between scanner, over all 4 scans. The COVs were larger for MTR_asym_ compared for those of LD and MTR_REX_, and for all three metrics COVs were mostly lower for within-session measurements compared to measurements between sessions and scanners.

**Table 2:** Group median (interquartile range Q1–Q3) of COV for within-session, between-session and between-scanner analysis per ROI per APT-weighted CEST metric.

COV of LD (%)		WM	GM	CSF
	Within session (N = 19)	0.56 [0.20–1.01]	0.68 [0.49–1.48]	3.04 [1.49–5.68]
	Between session (N = 6)	0.84 [0.38–1.27]	1.52 [0.72–2.41]	4.76 [3.74–6.70]
	Between scanner (N = 9)	0.80 [0.48–1.26]	1.28 [0.77–3.34]	6.62 [6.27–8.38]
	All sessions (N = 6)	1.91 [1.08–3.17]	3.96 [2.30–7.13]	7.96 [6.16–8.33]

COV of MTR_asym_ (%)		WM	GM	CSF
	Within session	2.62 [0.94–7.08]	2.64 [1.35–7.00]	19.62 [7.14–35.90]
	Between sessions	4.58 [3.48–9.66]	13.35 [9.40–19.99]	18.43 [3.86–47.32]
	Between scanner	10.46 [2.69–15.72]	57.59 [32.81–68.53]	8.37 [3.27–31.11]
	All sessions	12.22 [8.55–36.17]	49.47 [36.71–53.18]	35.28 [28.29–42.96]

COV of MTR_REX_ (%)		WM	GM	CSF
	Within session	1.28 [0.96–2.33]	2.34 [1.27–4.26]	3.94 [2.33–5.92]
	Between sessions	0.79 [0.24–2.26]	4.48 [3.28–7.44]	9.98 [6.38–10.69]
	Between scanner	9.30 [6.27–9.95]	8.85 [5.20–19.61]	10.98 [6.93–13.00]
	All sessions	8.67 [6.98–9.57]	21.00 [15.33–26.65]	17.40 [13.87–18.58]

**Figure 2:**
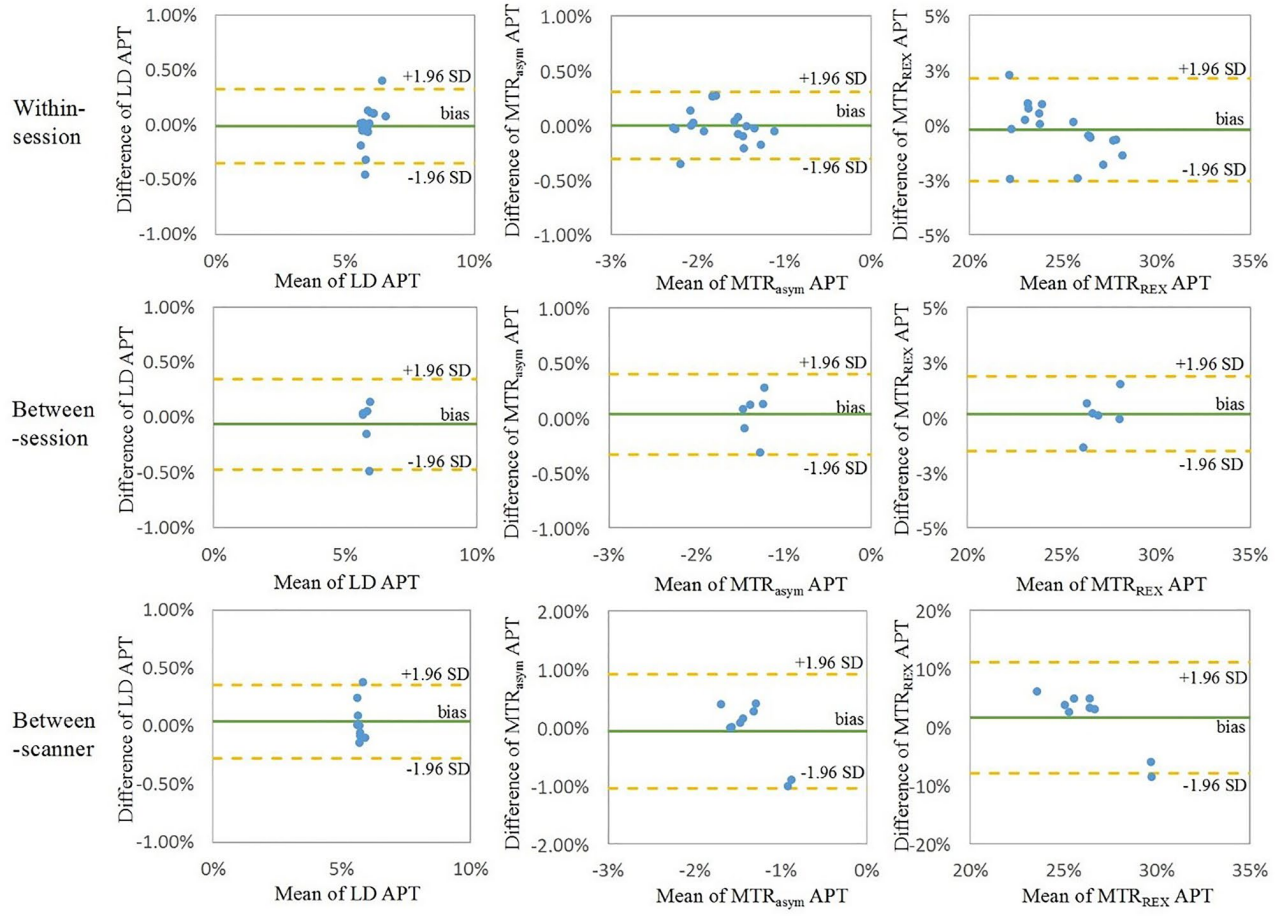
Bland–Altman plots of average APT values in WM from each participant. It shows the reproducibility of three CEST metrics in WM within-session, between-session and between-scanner.

The Bland–Altman plots of LD APT, MTR_asym_ and MTR_REX_ APT in WM showed similar standard deviations for the within-session and between-session analyses (Fig. 2). Both MTR_asym_ and MTR_REX_ APT showed larger standard deviations for between-scanner than for within-session measurements and between-session measurements.

The mixed effects analysis showed no significant effect of within-session, between-session or between-scanner variation on APT-weighted CEST measurement evaluated by either three metrics in the different ROIs.

### Patients

**Figure 3:**
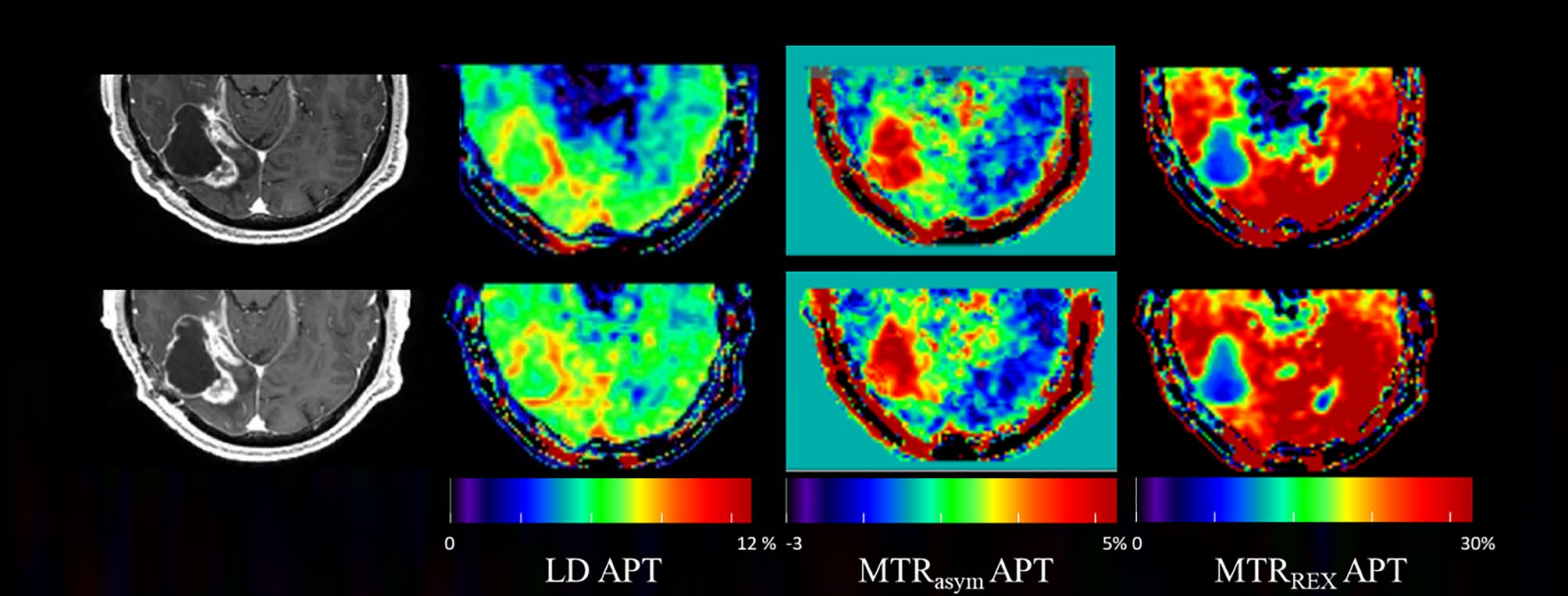
Example slice of patient 4 (recurrent glioblastoma in parieto-occipital lobe) showing from left to right, post contrast T_1_-weighted, LD APT, MTR_asym_ APT, and MTR_REX_ APT images from scan 1.a.1 and scan 2.a.1 (Fig. 1) to illustrate between-session reproducibility.

An example of the APT effect visualized with different metrics across two sessions can be seen in Fig. 3. From visual inspection, LD and MTR_asym_ consistently showed hyperintensity in the region of (enhancing) tumor compared with contralateral healthy tissue across two scan sessions, while for MTR_REX_ hypointensity can be observed consistently in the tumor ROI compared with healthy tissue.

**Figure 4:**
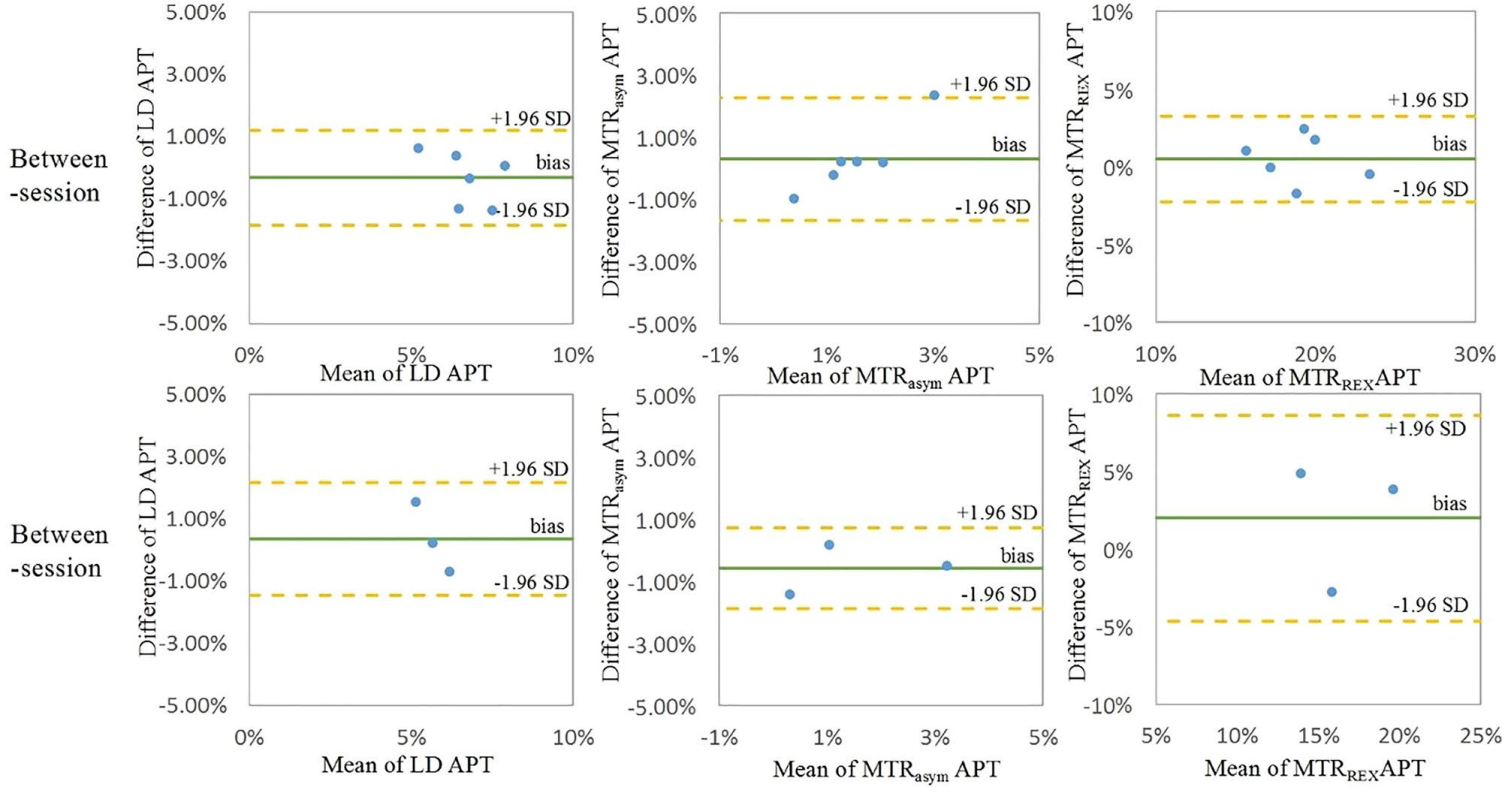
Bland–Altman plots of average APT values in CE tumor (top) and tumor core (bottom) from each participant. It shows the between-session reproducibility of three CEST metrics in tumor.

The APT effect in two scan sessions and COVs for the CE tumor and tumor core are shown in Table 3. LD APT and MTR_REX_ showed lower group median COV (4.57%, 3.89% respectively) than MTR_asym_ (9.20%) in tumor CE. The Bland–Altman plots show the deviations of APT-weighted CEST in tumor CE and tumor necrotic core measured by three metrics across two sessions (Fig. 4). The result of patient 3 was discarded because the size of tumor was too small for the CEST imaging spatial resolution, such that required downsampling during registration to CEST space, no voxels from the tumor mask remained. Patients 4,6 and 7 included in our study had a very small necrotic core region, such that the area(s) were too small to register into CEST space and could thus not be assessed separately.

**Table 3:** Group median [Q1–Q3] of the APT effect per scan session and between-session COV per ROI per metric.

	CE tumor (N = 6)			Tumor core (N = 3)		
	APT- weighted values in Session 1 (%)	APT- weighted values in Session 2 (%)	COV (%)	APT- weighted values in Session 1 (%)	APT- weighted values in Session 2 (%)	COV (%)
LD	6.61 [6.01–6.77]	7.04 [6.38–7.66]	4.57 [2.57–8.36]	5.88 [5.82–6.77]	5.92 [5.26–6.34]	5.67 [3.78–10.36]
MTR_asym_	1.53 [1.11–2.03]	1.35 [1.18–1.76]	9.20 [7.00–31.39]	2.06 [0.75–3.09]	1.37 [1.01–2.15]	8.93 [8.28–26.58]
MTR_REX_	19.24 [17.38–20.74]	18.56 [17.43–19.54]	3.89 [1.57–4.54]	18.96 [15.88–23.70]	15.05 [12.54–17.34]	9.78 [9.27–13.60]

The COV of each participant was computed across two sessions performed on different days.

## Discussion

In this work, we evaluated the repeatability and reproducibility of APT-weighted CEST imaging at 3 Tesla. This study was performed within and between sessions, and between two different scanners. The repeatability of the APT effect within a session was consistently better than the reproducibility between sessions and between scanners. The majority of COV values in our study were < 30%. In the comparison across three CEST metrics, LD and MTR_REX_ provided more robust measurement than MTR_asym_ with COV < 10%, both in healthy volunteers and patients, as illustrated by smaller COVs.

To interpret COV, we refer to a grading scheme introduced previously for hepatic perfusion imaging biomarkers^46^ where COV < 10% is considered very good, 10% < COV < 20% as good and 20% < COV < 30% as acceptable. There was a trend of increasing COV from within-session to between-session and between-scanner for each CEST metric tested, going from very good within-session repeatability to good or acceptable between-session and between-scanner reproducibility. This is likely explained by increasing variability between measurements done on separate days and separate scanners. For instance, in two sessions across one week, differences in body temperature and physiological level of protein can influence the magnitude of the CEST effect by affecting the fractional concentration of the solute protons^2^, as for instance shown in the liver after fasting^47^. However, in particular for the brain where fluctuations in physiology are expected to be limited, stronger effects on CEST signal are likely caused by differences in participant positioning (compared to the iso-center of the system, and leading to differences in shimming of the field of view) and scanner state or between-scanner set-ups. The different scanners included different head coils and system versions (32 channel coil and DV26 software environment for the MR750 scanner, 24 channel coil and MP26 software environment for the PET-MRI scanner). Moreover, the PET-MRI scanner has a smaller bore size, due to the presence of the PET-detectors. All this can influence the signal-to-noise ratio of images, and B_0_ and B_1_ fields, leading to differences in evaluated APT-weighted effects. Based on the higher COV of between-scanner versus between-session reproducibility experiments indeed confirm that the influence of these scanner differences seems to be larger than body conditions.

In comparing the three different APT-weighted CEST metrics, we found the smallest COV for LD APT, which consistently showed very good repeatability and reproducibility (all COV < 10%). This finding is in line with a previous study where COV < 10% within session in WM and GM was reported^14^. MTR_REX_ provided good repeatability and reproducibility (< 20%) in most experiments. Small COV and better consistency in LD/MTR_REX_ APT compared to MTR_asym_ is likely a result of the way these APT metrics are computed. During the calculation of MTR_asym_, residual direct water saturation, magnetization transfer effects and nuclear Overhauser effects are not compensated for. Signal variation coming from those effects can decrease the repeatability/reproducibility of the MTR_asym_. The magnitude of MTR_asym_ is usually smaller than LD and close to 0 (in tumor ~ 2%, healthy tissue ~ -1%). This effectively reduces SNR, which may be contributing to the additional variation of MTR_asym_ across sessions, compared to LD/MTR_REX_. Both LD and MTR_REX_ were calculated by subtracting a two-pool fitted Z-spectrum, in which DS and MT were fitted, from the acquired Z-spectrum data. The APT effects evaluated by LD and MTR_REX_ were calculated based on this subtracted Z spectrum at 3.5 ppm only, such that the data acquired at -3.5 ppm was not included hereby avoiding NOE effects in the amide-weighted metrics. This approach of calculating LD and MTR_REX_, with minimizing effects of DS, MT and NOE, may be the reason for these metrics providing more consistent measurements than MTR_asym_.

In patients, the COV of MTR_asym_ reported here is comparable with a recent study where 11–30% COV of MTR_asym_ was found in glioma in between-session experiments, which is considered to fall within acceptable reproducibility^29^ for quantifying and monitoring glioma. Note that both in our healthy volunteer and patient study, much lower COV for LD and MTR_REX_ was found. For quantitative APT-weighted imaging in clinical practice, a small change of APT effect can impact the ability to differentiate tumors, such as an MTR_asym_ difference of 0.5% between solitary brain metastasis and glioma^48^, and an MTR_asym_ difference of 0.4% in the prediction of isocitrate dehydrogenase (IDH) mutation status in grade II gliomas^49^. We found between session differences in MTR_asym_ APT to be ~ 0.3%. Thus, the use of advanced metrics LD/MTR_REX_ is preferable for using APT-weighted CEST MRI in diagnosis, not only as these metrics have higher reproducibility, but also because they are likely able to provide better differentiation between tumors in clinical practice.

Additionally, with using LD/MTR_REX_ it is feasible to investigate the changes in MTR at 3.5 ppm and − 3.5 ppm independently. While detection of increased amide-weighted MTR at 3.5 ppm has been of main focus for brain tumor imaging, evidence for decreases in NOE-weighted MTR in high grade brain tumors is increasingly reported and potentially useful for diagnostics and treatment follow-up^3,6,50^. This gives another reason why the use of MTR_asym_ in brain tumor diagnostics may be suboptimal.

Another promising clinical application of APT-weighted CEST MRI is in early detection of true tumor progression after treatment of high grade gliomas. The COV found here for all three metrics are likely sufficient for this purpose, even for MTR_asym_. There are mostly retrospective or cross-sectional studies that have investigated ATPw-CEST MRI at a single time point after progression. The difference found between true progression and treatment effect is at minimum 200% (a two-fold higher value) in MTR_asym_ for true progression in these retrospective, cross-sectional studies^51,52^. Moreover, in one longitudinal data set (albeit a small cohort) it is reported that there is stable MTR_asym_ signal in progressive disease after surgery and radio- and chemotherapy with tumor averages of MTR_asym_ after treatment varying between 3.5 and 7% compared to pre-treatment values, whilst there is an almost 70% decrease in MTR_asym_ values for patients with treatment effect^53^. Our COV values for LD (between-session COV of approximately 5% for contrast enhancing tumor and 7.5% for tumor core) and even for MTR_asym_ (20–24%) would still be sufficient to detect treatment related changes with the above expected effect size of, at minimum, 70%.

It should be noted that MTR_asym_ can be acquired by obtaining only few off-resonance images making acquisition inherently faster, and more attractive for clinical application, than the need for acquiring a full z-spectrum required for LD and MTR_REX_ calculations. This is why in the consensus on application of CEST MRI for brain tumor imaging, MTR_asym_ currently is recommended^9^. However, this consensus recommendation includes doing B_0_ field inhomogeneity correction, either with a separate, fast acquisition or within the CEST acquisition because of the detrimental effect B_0_ fluctuations have on MTR_asym_. While this inherently adds to the acquisition time, advances in image acquisition and analysis are leading to more rapid scan times, not only enabling MTR_asym_ acquisition with B0 correction, but also LD/MTR_REX_ acquisition to become clinically feasible, as exemplified in our current scanning protocol, where all three metrics and B_0_ correction can be obtained within one single, volumetric scan of fewer than 5 min acquisition time.

In this work, we performed between-scanner reproducibility measurements and provided evidence that the APT-weighted CEST effect can be reproduced well on two scanners. We also showed the feasibility of providing consistent measurements in patients with brain tumors. However, we only included scanners from a single vendor, and our patient cohort was fairly small and heterogeneous. Due to wanting to keep the burden to our patient cohort low, we did not include between-scanner measurements for the patients. Moreover, we cannot fully rule out that there were changes in tumor physiology between the two measurements within the patients because of the highly proliferative nature of high grade tumors. Our future work is aimed at assessing repeatability and reproducibility of scanners from different vendors and at different hospitals, while extending the patient cohort. In particular when it comes to comparing between scanners from different vendors, it will be important to investigate effects from the unavoidable deviations in acquisition parameters and hardware. Such further assessments are essential for the field to deliver good between-session and between-scanner reproducibility, such that APT-weighted CEST can eventually become a quantitative imaging biomarker for clinical practice and multi-centre research trials including brain tumor imaging.

## Conclusion

In summary, our study provides further evidence that APT-weighted CEST imaging is repeatable and reproducible in healthy brain and brain tumors across scan sessions and scanners at 3 Tesla. While MTR_asym_ provides acceptable reproducibility, more advanced metrics (LD and MTR_REX_) show much better reproducibility which is of importance when subtle differences in APT-weighted CEST are sought for clinical diagnosis or monitoring of brain pathology. Future work in translating APT-weighted CEST MRI for clinical application in brain tumor diagnostics should include measuring reproducibility across different sites and different vendors to confirm APT-weighted CEST as a reproducible and quantitative imaging biomarkers for brain tumor imaging.

### Supplementary Information

Supplementary Information.

## Data Availability

The data that support the findings of this study are available from the corresponding author upon request.
