# Supplementary material for: Reproducibility of APT-weighted CEST-MRI at 3T in healthy brain and tumor across sessions and scanners
Source: Sci Rep. 2023 Oct 23;13:18115. doi: 10.1038/s41598-023-44891-0 (PMC10593824; doi:10.1038/s41598-023-44891-0)
Supplement: Supplementary file 1 — Supplementary Information. [file 41598_2023_44891_MOESM1_ESM.docx]

**Appendix**

To calculate the COV we used the following equations for the healthy volunteer data set:

$${COV}_{within}=\frac{\sigma_{A_{ROI,1.a.1},A_{ROI,1.a.2}}}{0.5(A_{ROI,1.a.1}+A_{ROI,1.a.2})}$$

$${COV}_{between session}=\frac{\sigma_{A_{ROI,1.a.1},A_{ROI,2.a.1}}}{0.5(A_{ROI,1.a.1}+A_{ROI,2.a.1})}$$

$${COV}_{between scanner}=\frac{\sigma_{A_{ROI,1.a.1},A_{ROI,1.b.1}}}{0.5(A_{ROI,1.a.1}+A_{ROI,1.b.1})}$$

Where A stands for the CEST metric of interest, i.e. LD/MTR_asym_/MTR_REX_, and ROI stands for the ROI of interest, i.e. WM/GM/CSF.

To calculate the COV we used the following equations for the patient data set:

$${COV}_{between session}=\frac{\sigma_{A_{ROI,1.b.1},A_{ROI,2.b.1}}}{0.5(A_{ROI,1.b.1}+A_{ROI,2.b.1})}$$

Where A stands for the CEST metric of interest, i.e. LD/MTR_asym_/MTR_REX_, and ROI stands for the ROI of interest, i.e. WM/tumor core/contrast enhancement.
